# Assessing the applicability of Society of Thoracic Surgeons–European Association for Cardio-Thoracic Surgery (STAT) categories in neonatal and low-weight congenital cardiac surgery: Insights from 20 years at a single center

**DOI:** 10.1016/j.xjon.2026.101843

**Published:** 2026-04-28

**Authors:** Eitan Keizman, Alain Serraf, Evyatar Hubara, Reut Kassif-Lerner, David Mishaly, Uri Pollak

**Affiliations:** a Division of Cardiac Surgery, Columbia University Vagelos College of Physicians and Surgeons, Morgan Stanley Children's Hospital of NewYork-Presbyterian, New York, NY; b The Edmond J. Safra International Congenital Heart Center, Sheba Medical Center, Tel Hashomer, Israel; c Gray Faculty of Medical & Health Sciences, Tel Aviv University, Tel Aviv, Israel; d Department of Pediatric Cardiac Surgery, Hadassah University Medical Center, Jerusalem, Israel; e Faculty of Medicine, The Hebrew University of Jerusalem, Jerusalem, Israel; f Section of Pediatric Critical Care, Hadassah University Medical Center, Jerusalem, Israel

**Keywords:** congenital cardiac surgery, neonatal repair, cardiopulmonary bypass, STAT mortality score

## Abstract

**Objective:**

The Society of Thoracic Surgeons–European Association for Cardio-Thoracic Surgery (STAT) Mortality Categories were developed from large multicenter databases to stratify surgical risk in congenital heart surgery. However, their predictive validity in high-risk subgroups such as neonates and infants of low birth weight, especially in small- to medium-volume centers, remains unclear.

**Methods:**

We retrospectively analyzed all congenital cardiac surgeries performed at our center between 2005 and 2025. We assessed 30-day mortality by STAT category in the overall cohort and in prespecified subgroups: neonates (<31 days), non-neonates (≥31 days), and low birth weight (<2.5 kg). We performed correlation analyses and multivariable logistic regression models with interaction terms (STAT × neonate, STAT × low weight).

**Results:**

Among 4768 operations, 1257 (26.3%) were in neonates and 191 (4.0%) in patients <2.5 kg. Overall mortality increased progressively with STAT category (1: 1.1%, 2: 3.7%, 3: 5.8%, 4: 12.8%, 5: 26.7%). Neonates exhibited greater mortality across all STAT levels, including categories considered low risk in older children (STAT 1: 9%, STAT 2: 6%). Logistic regression showed that STAT was a strong predictor of mortality (odds ratio [OR], 2.26; 95% CI, 1.94-2.64), whereas neonates had a greater baseline risk (OR, 3.88; 95% CI, 1.60-9.40); however, the STAT × neonate interaction suggested attenuated predictive performance in neonates (OR, 0.80; 95% CI, 0.63-1.01).

**Conclusions:**

STAT categories were associated with mortality overall but demonstrated attenuated discrimination in neonates, highlighting the limitations of procedure-only stratification in physiology-dominated subgroups.

**Figure:**
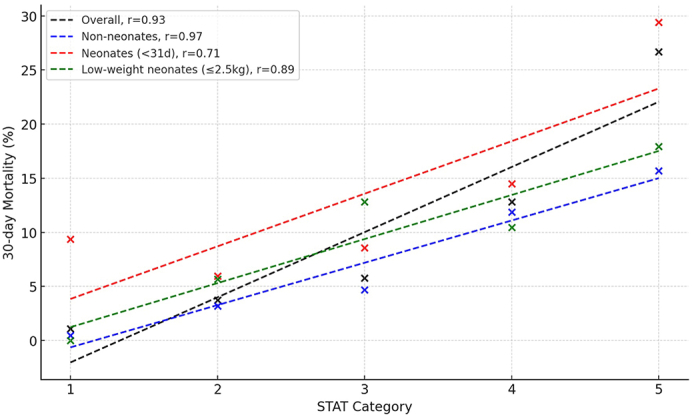
STAT-mortality correlation weakens in neonates and infants of low weight.

**Central Message**

STAT categories predict mortality well overall, but discrimination is markedly attenuated in neonates and infants of low weight, highlighting the need for patient-level risk refinement.

**Perspective**

This study shows that although STAT categories perform well for most congenital cardiac operations, their discriminatory accuracy weakens in neonates and infants of very low weight. These findings highlight the limitations of procedure-only risk models in physiology-dominated subgroups and support integrating simple patient-level variables to improve risk estimation and benchmarking.

Congenital and pediatric cardiac surgery has undergone remarkable progress over the past 5 decades, driven by advances in surgical techniques, perioperative care, and multidisciplinary collaboration. This field involves highly specialized teams caring for one of the most complex and fragile patient populations in modern medicine.^1^ As such, risk stratification is central to the evaluation of congenital cardiac surgery outcomes.^2^ The Society of Thoracic Surgeons–European Association for Cardio-Thoracic Surgery (STAT) Mortality Categories were derived from pooled multicenter databases of the Society of Thoracic Surgeons (STS) and European Association for Cardio-Thoracic Surgery (EACTS), encompassing tens of thousands of procedures across high-volume centers. These categories group operations by their empirically observed mortality, enabling intercenter comparison and quality benchmarking.^2^, ^3^, ^4^, ^5^

Before the development of the STAT Mortality Categories, surgical complexity in congenital heart surgery was commonly assessed using the Aristotle Basic Complexity Score.^6^ Although the Aristotle score reflected expert consensus regarding technical difficulty, it was not determined on the basis of observed outcome data. STAT, in contrast, was derived empirically from large multicenter datasets and has largely replaced Aristotle as the preferred system for risk stratification.^4^^,^^6^ However, the STAT Mortality Categories were designed as a procedure-based risk stratification framework derived from empirically observed mortality, rather than a comprehensive patient-level risk adjustment model. Accordingly, STAT categories are well suited for case-mix description and stratified reporting, but do not, by themselves, provide the covariate-rich adjustment required for center-level comparisons. Contemporary STS benchmarking therefore relies on multivariable mortality risk models that incorporate STAT (or related procedure risk) alongside key patient-level predictors. As such, the STAT categories do not incorporate patient-level factors such as age, weight, prematurity, or organ immaturity, parameters that are particularly impactful in cardiac surgery.^3^ Neonates (<31 days) and especially neonates with low birth weight (<2.5 kg) represent a uniquely vulnerable population with a well-documented increased risk of surgical morbidity and mortality.^7^ Even in high-volume centers, mortality in this group has been reported between 15 and 25%, which is substantially greater than in older children.^8^^,^^9^ Factors such as small vessel size, longer cardiopulmonary bypass (CPB) times, increased hemodilution, and difficulty in postoperative recovery contribute to this elevated risk.^10^ These factors may dilute the predictive value of procedure-based risk models like STAT, raising concern that the system might underestimate mortality risk in these fragile patients.^11^

In addition, although centers contributing to the STS Congenital Heart Surgery Database vary widely in annual surgical volume, the relative contribution of individual centers is not publicly available. STAT Mortality Categories are therefore intentionally derived from the aggregate database rather than from volume-stratified subsets, ensuring that procedure-based risk stratification reflects contemporarily observed outcomes across the full spectrum of participating programs.^12^ Understanding how well STAT predicts outcomes in the settings of small- or medium-volume centers is therefore clinically relevant. The objective of this study was to evaluate the association between STAT category and early mortality in a 20-year single-center cohort, with specific focus on neonates and infants with low birth weight (Figure 1).

**Figure 1:**
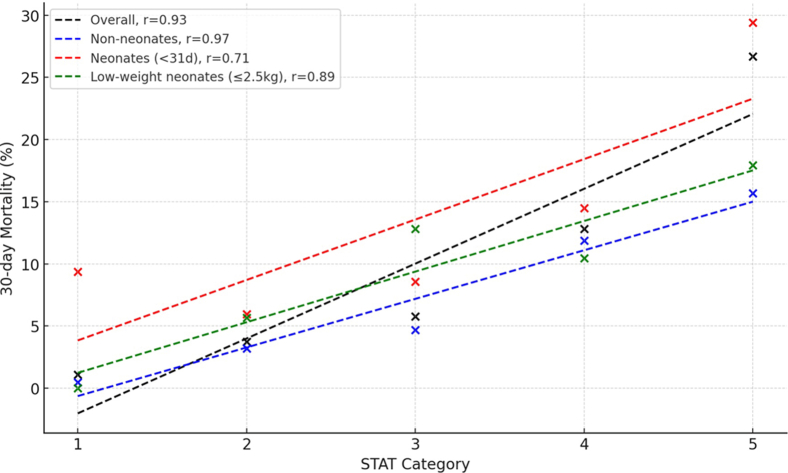
STAT mortality correlation weakens in neonates and infants with low weight. S*TAT*, Society of Thoracic Surgeons–European Association for Cardio-Thoracic Surgery Mortality Categories.

## Methods

### Study Setting and Population

We retrospectively reviewed all congenital cardiac surgeries performed at our institution between January 2005 and February 2025. The study included all surgical procedures for which a STAT category was defined. Procedures without an assigned STAT category were excluded from the analysis. These included operations that are not classified within the STAT framework, such as ventricular assist device implantation, surgical extracorporeal membrane oxygenation cannulation, delayed sternal closure, diaphragm plication, and more. As a result, the number of operations included in the present analysis is lower than the total number of congenital cardiac procedures performed during the study period.

**Figure 2:**
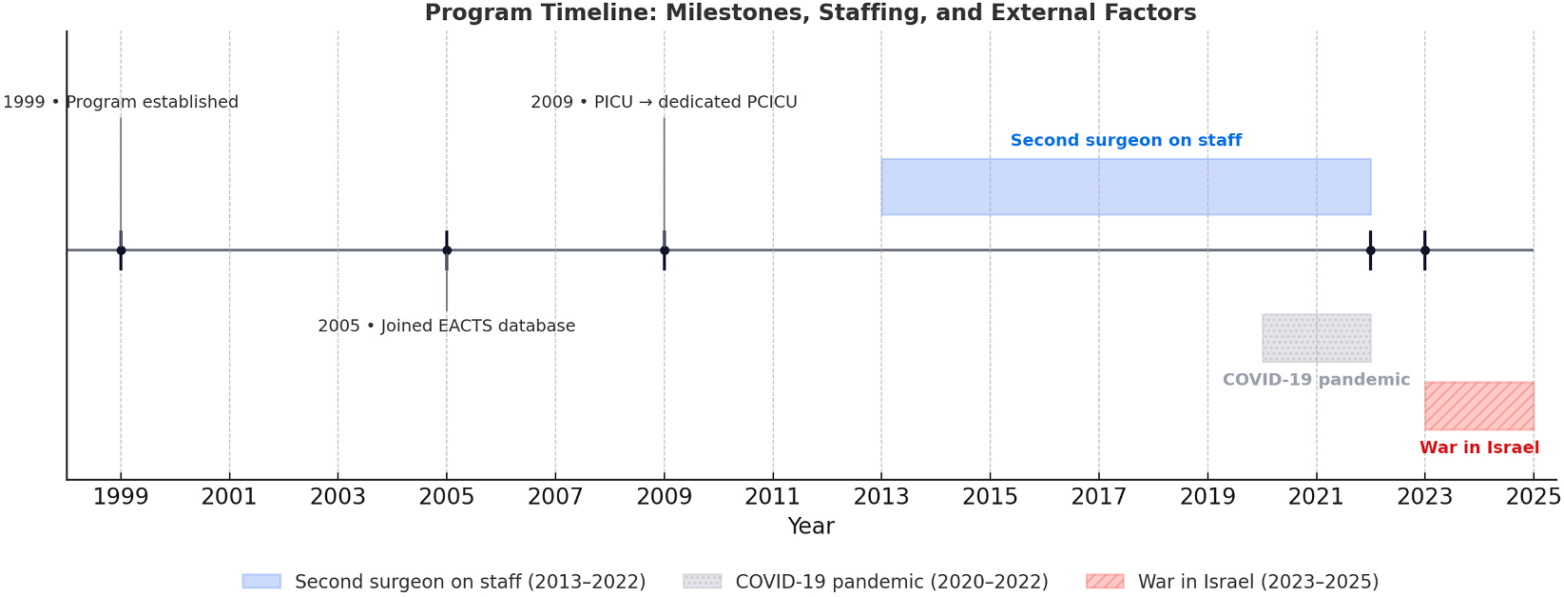
Program timeline and key milestones (1999-2025). The congenital cardiac surgery program was established in 1999 and joined the EACTS database in 2005 under a single primary congenital surgeon. In 2009, postoperative care was reorganized with the creation of a dedicated pediatric cardiac intensive care unit (*PCICU*). Surgical capacity expanded in 2013 with the recruitment of a second congenital surgeon, who remained through 2022, after which the service returned to a single-surgeon model. From 2020 to 2022, the COVID-19 pandemic affected volumes and case mix. In 2023, war in Israel abruptly halted referrals from the Gaza Strip—an especially consequential change because, before the war, Palestinian infants and children from Gaza accounted for roughly one-third of our annual surgical volume. Referrals from the West Bank and East Jerusalem, however, have continued on an ongoing basis, preserving the program's regional referral role despite the loss of Gaza cases. *EACTS*, European Association for Cardio-Thoracic Surgery; *PICU*, Pediatric intensive care unit.

Our center has maintained a modest annual volume of congenital cardiac operations. Over the years, several charitable organizations, in collaboration with Sheba Medical Center, have contributed to the funding of medical and surgical care for infants and children with congenital heart disease at our institution. Until October 2023, approximately one third of the surgical volume consisted of referrals from the Gaza Strip; although these referrals have since ceased, patients from the West Bank continue to be referred on an ongoing basis. In addition, we have treated a substantial number of patients from Albania, Ukraine, Syria, Kazakhstan, and Nigeria. Several notable milestones have shaped the evolution of the program over the past 2 decades and are presented in Figure 2.

The study presents operations rather than the number of patients, because each patient could contribute more than 1 operation. This approach was chosen because many patients underwent multiple, temporally distinct surgical procedures across separate hospitalizations (eg, staged palliation with Norwood, Glenn, and Fontan operations), each representing an independent operative exposure and risk period; such operations were therefore included separately in the analysis. In contrast, when multiple procedures were performed during the same operative episode and hospitalization, the operation was treated as a single index case, and the procedure corresponding to the primary indication for admission was used for classification, consistent with STS Congenital Heart Surgery Database methodology. Operations were categorized as CPB-assisted and non–CPB-assisted operation. Neonates were considered age <31 days at surgery,^13^ and patients with low weight were considered those at body weight <2.5 kg at the day of surgery, constituted a subset of this neonatal population, and were analyzed as a prespecified physiologically defined subgroup, reflecting the particular clinical vulnerability associated with very low body weight. STAT Mortality Category (1-5) was assigned to each operation according to the most recent published STAT classification. When multiple procedures were performed during a single hospitalization, mortality was attributed to a single index operation for that episode of care, defined as the procedure corresponding to the primary indication for which the patient was admitted. In practical terms, the STAT category was assigned according to the operation representing the main therapeutic objective of the admission. This approach follows the general principle used in the STS Congenital Heart Surgery Database of assigning outcomes to the primary procedure of the operative episode while acknowledging that full retrospective application of the detailed STS primary-procedure adjudication algorithm was not feasible in our dataset. The primary outcome was 30-day all-cause mortality, defined as death occurring (1) during the hospitalization regardless of postoperative day, (2) after hospital discharge but within 30 days of surgery, or (3) after 30 days if the patient remained continuously hospitalized from the index operation.

Data were extracted from the center's EACTS online records. Cases were entered into the EACTS online database only after a minimum of 30 days after hospital discharge, to ensure complete ascertainment of 30-day mortality. We extracted age, weight, sex, diagnosis, type of surgery, CPB use (yes/no), CPB and crossclamp times, and 30-day mortality.

The study was approved by the hospital institutional review board of Sheba Medical Center (approval: 3976-17. date: April 6, 2025). Supporting data are available upon reasonable request.

### Statistical Analysis

Descriptive analyses summarized demographics and outcomes across predefined groups (neonates vs non-neonates, low-weight [<2.5 kg] vs non-low-weight). Pearson correlation between STAT category and 30-day mortality was initially computed as a descriptive measure of monotonic association for the overall cohort and for each subgroup (neonates, non-neonates, and patients with low weight). However, given that correlation alone does not quantify predictive performance, model discrimination and calibration were formally evaluated. Because STAT category is an ordinal variable and mortality is a binary outcome, discrimination was additionally evaluated using the C-statistic (area under the receiver operating characteristic curve), which provides a standard measure of the ability of increasing risk categories to discriminate mortality. Discriminative ability was assessed using the area under the receiver-operating characteristic curve (AUC) with 95% CIs estimated by using the method from DeLong. Calibration performance was evaluated through the Brier score, calibration-in-the-large (intercept), and calibration slope. For descriptive mortality estimates across STAT categories, 95% CIs were calculated using the exact binomial (Clopper-Pearson) method.

Era-stratified analyses were performed to account for temporal changes in program structure and case mix. The study cohort was divided into 4 periods (2005-2009, 2010-2014, 2015-2019, and 2020-2025). Within each era, correlations between STAT category and 30-day mortality were assessed separately for neonates, non-neonates, and infants with low weight (<2.5 kg). Logistic regression models including interaction terms (STAT × era, neonate × era) were used to evaluate whether risk discrimination and calibration varied over time. A multivariable logistic regression model was constructed to examine the independent association between STAT category and 30-day mortality. The model included STAT category (ordinal), neonatal status, low birth weight status, and the prespecified interaction terms (STAT × neonatal status; STAT × low weight). Intraoperative variables such as CPB use, bypass duration, and aortic crossclamp time were not included, because they lie on the causal pathway from procedural complexity to outcomes. Era effects were evaluated separately using era-stratified models and interaction terms. Odds ratios (ORs) with 95% CIs were reported.

In addition, an observed-to-expected (O/E) analysis was performed using established STAT-band mortality benchmarks from recent STS harvest reports. Expected deaths were estimated for each STAT category by multiplying the corresponding benchmark mortality by the number of eligible cases. O/E ratios with exact binomial 95% CIs were computed for the entire cohort, neonates, and patients with low weight. These O/E values were subsequently integrated with the calibration analysis to assess whether deviations from predicted risk were systematic (under- or overprediction) across eras and patient strata. Expected mortality was estimated using published STAT category–specific mortality benchmarks from recent STS Congenital Heart Surgery Database harvest reports^5^ and applied uniformly across the study period as an external reference.

All statistical tests were 2-sided. Continuous variables were compared using the *t* test or Mann-Whitney *U* test, and categorical variables were compared using the Fisher exact test or χ^2^ test, as appropriate. Analyses were conducted using Python (version 3.11.6).

## Results

### Cohort Characteristics

Since we joined the EACTS database, a total of 5445 operations have been performed at our institution, of which 4768 were included in the present analysis, performed in 3945 individual patients over the study period of 2 decades. Of these, 1257 operations (26.3%) were performed in neonates (<31 days of age), and 191 operations (4.0%) involved patients weighing less than 2.5 kg at the time of surgery. The majority of operations were CPB-assisted, accounting for 88% of all operations and 93% of those in neonates. The overall cohort had a median age at operation of 1.2 years (interquartile range, 0.05-3.8 years) and a median weight of 6.1 kg (interquartile range, 3.1-13.2 kg). The overall 30-day mortality rate was 8.9% and the mean STAT category across the entire cohort was 2.6±1.2. Data regarding the number of patients in each group, weight, CPB, and crossclamp times are stratified by group and STAT and presented in Table 1.

**Table 1:** Patient characteristics

Category	Metric	Low weight	Neonates	Non-neonates	All
Stat 1	Eligible	33	64	840	904
	Mortality, n	0	6	4	10
	Mortality, %	0	9.4	0.5	1.1
	On CPB, n, %	8, 24.2	35, 54.7	651, 77.5	686, 75.9
	Median CPB time (IQR)	0.0 (41.5)	46.0 (27.2)	44.0 (27.0)	44.0 (27.0)
	XCP time n, %	8, 24.2	35, 54.7	409, 48.7	444, 49.1
	Median XCP time (IQR)	0.0 (32.0)	33.0 (20.0)	26.0 (37.8)	27.0 (38.8)
	Weight, median (IQR)	1.35 (1.29)	3.0 (1.01)	11.0 (14.2)	10.5 (14.0)
Stat 2	Eligible	64	268	1121	1389
	Mortality, n	3	16	36	52
	Mortality, %	4.7	6	3.2	3.7
	On CPB, n, %	14, 21.9	72, 26.9	893, 79.7	965, 69.5
	Median CPB time (IQR)	0.0 (83.0)	11.0 (54.5)	63.0 (49.5)	60.0 (52.0)
	XCP time n, %	13, 20.3	57, 21.3	608, 54.2	665, 47.9
	Median XCP time (IQR)	0.0 (62.0)	0.0 (33.0)	48.0 (53.0)	41.5 (68.0)
	Weight, median (IQR)	2.0 (0.5)	3.0 (0.7)	10.0 (11.3)	7.6 (10.9)
Stat 3	Eligible	35	245	640	885
	Mortality, n	6	21	30	51
	Mortality, %	17.1	8.6	4.7	5.8
	On CPB, n, %	34, 97.1	231, 94.3	585, 91.4	816, 92.2
	Median CPB time (IQR)	106.5 (65.0)	110.5 (65.8)	84.0 (56.5)	89.0 (61.0)
	XCP time n, %	32, 91.4	226, 92.2	376, 58.8	602, 68.0
	Median XCP time (IQR)	81.0 (60.0)	87.0 (58.5)	64.0 (52.0)	70.0 (59.0)
	Weight, median (IQR)	2.3 (0.3)	3.1 (0.75)	7.78 (7.4)	6.0 (6.5)
Stat 4	Eligible	92	476	859	1335
	Mortality, n	8	69	102	171
	Mortality, %	8.7	14.5	11.9	12.8
	On CPB, n, %	41, 44.6	225, 47.3	583, 67.9	808, 60.5
	Median CPB time (IQR)	53.0 (85.0)	47.5 (86.0)	75.0 (90.5)	66.0 (84.0)
	XCP time n, %	33, 35.9	161, 33.8	446, 51.9	607, 45.5
	Median XCP time (IQR)	27.0 (70.0)	29.0 (70.0)	56.0 (87.5)	47.0 (88.0)
	Weight, median (IQR)	2.2 (0.3)	3.04 (0.76)	5.9 (6.62)	4.0 (4.4)
Stat 5	Eligible	33	204	51	255
	Mortality, n	6	60	8	68
	Mortality, %	18.2	29.4	15.7	26.7
	On CPB, n, %	29, 87.9	185, 90.7	43, 84.3	228, 89.4
	Median CPB time (IQR)	114.0 (45.0)	107.0 (52.5)	104.0 (47.5)	105.0 (53.0)
	XCP time n, %	28, 84.8	180, 88.2	43, 84.3	223, 87.5
	Median XCP time (IQR)	73.5 (26.5)	69.0 (45.0)	67.0 (45.0)	68.5 (45.5)
	Weight, median (IQR)	2.2 (0.26)	3.0 (0.73)	6.05 (3.35)	3.1 (0.9)

This table summarizes eligible case counts, 30-day mortality, CPB use, CPB and crossclamp durations, and weight distribution across STAT mortality categories 1-5. Data are presented separately for infants with low weight (<2.5 kg), neonates (<31 days), non-neonates (≥31 days), and the overall cohort. Across categories, neonates and infants with low weight show consistently greater mortality rates and undergo CPB more frequently than non-neonates. CPB and crossclamp times increase with procedural complexity. Median patient weight demonstrates expected differences across strata and STAT categories. *CPB*, Cardiopulmonary bypass; *IQR*, interquartile range; *XCP*, aortic crossclamp; *STAT*, Society of Thoracic Surgeons-European Association for Cardio-Thoracic Surgery Mortality Category.

### Mortality by STAT Category

When stratified by STAT mortality category, 30-day mortality increased progressively across the entire cohort, from 1.1% in STAT 1, 3.7% in STAT 2, 5.8% in STAT 3, and 12.8% in STAT 4, up to 26.7% in STAT 5. Neonates exhibited consistently greater mortality across all STAT levels compared with non-neonates. Notably, neonatal mortality remained high even in categories generally considered low risk: 9% in STAT 1, 6% in STAT 2, and 8.6% in STAT 3, compared with 0.5%, 3.2%, and 4.7%, respectively, in non-neonates. At greater STAT levels, the gap persisted, with mortality reaching 14.5% versus 11.9% in STAT 4, and 29.4% versus 15.7% in STAT 5 among neonates versus non-neonates, respectively. The group of patients with low weight (<2.5 kg), regardless of age, showed a pattern resembling that of neonates: mortality was elevated even at the lowest STAT categories (STAT 1-2).

**Figure 3:**
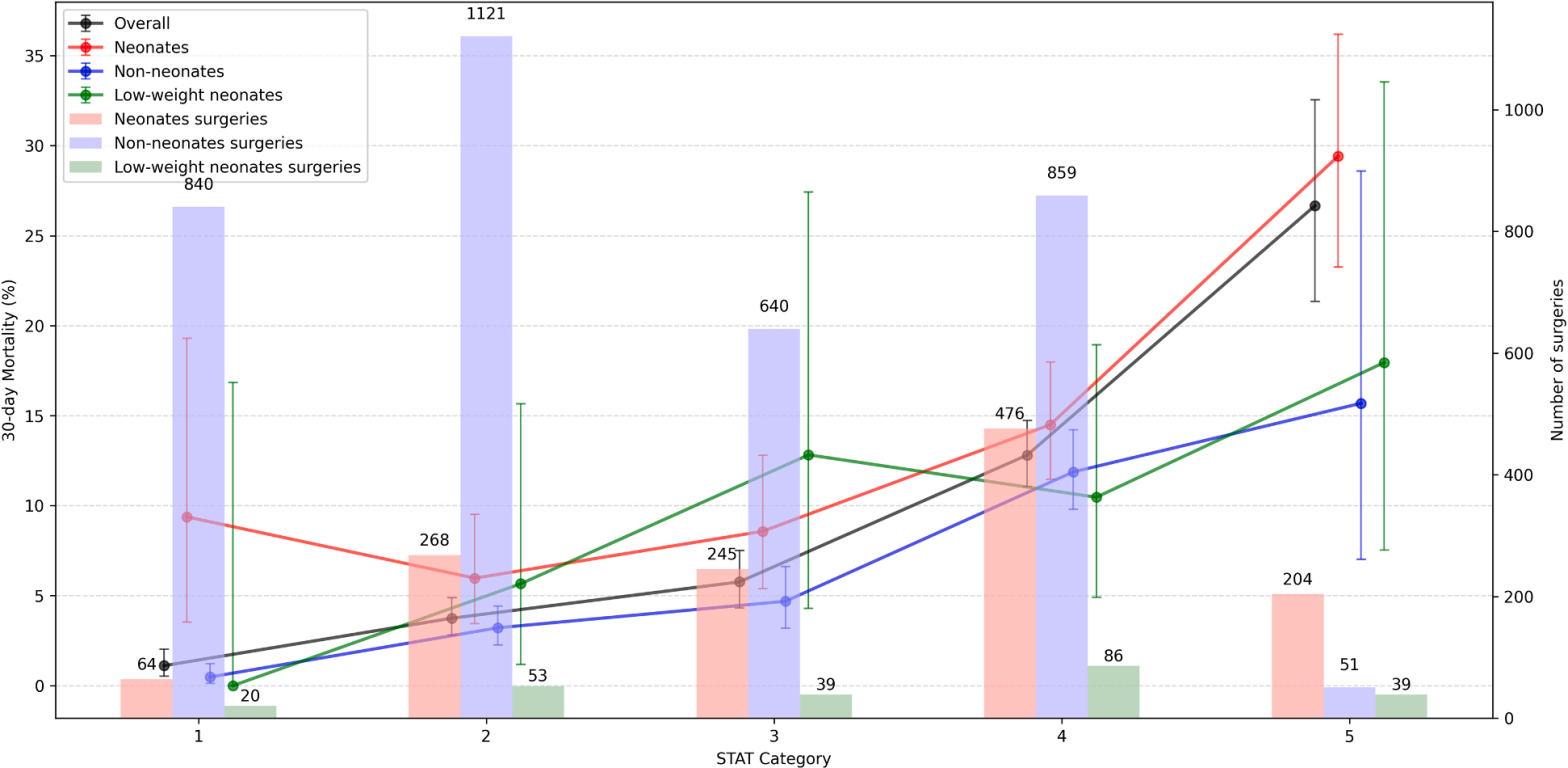
Thirty-day mortality across STAT mortality categories in the overall cohort and in prespecified subgroups. The figure displays how 30-day operative mortality increases monotonically with STAT mortality category in the overall population, with *parallel curves* shown for neonates (<31 days), non-neonates, and neonates with low weight (<2.5 kg). *Error bars* indicate 95% CIs, calculated using the exact binomial method. Despite the expected stepwise increase in mortality with increasing STAT level, neonates demonstrate substantially greater mortality at every category, including those traditionally regarded as low risk (STAT 1-2). Neonates with low weight show a similar pattern of disproportionate mortality, particularly in midrange categories. These findings illustrate attenuation of STAT's discriminatory performance in physiology-dominated strata, in which patient vulnerability contributes more strongly to early postoperative risk than procedural complexity alone. *STAT*, Society of Thoracic Surgeons–European Association for Cardio-Thoracic Surgery Mortality Categories.

Discrimination analysis using the C-statistic demonstrated moderate overall discrimination (AUC, 0.73), with greater performance in non-neonates (AUC, 0.74) and reduced discrimination in neonates (AUC, 0.66) and infants of very low weight (AUC, 0.65). These trends are illustrated in Figure 3.

Overall, an O/E mortality analysis was performed using STAT-band benchmark operative mortality rates derived from the STS Congenital Heart Surgery Database.^5^ Expected deaths were estimated for each STAT category by multiplying the corresponding benchmark mortality by the number of eligible cases and summing across categories. Overall, the O/E ratio for 30-day mortality was 1.71 (95% CI, 1.55-1.90). In neonates, the O/E ratio was 1.98 (95% CI, 1.71-2.27) and in patients with low birth weight (<2.5 kg) was 1.47 (95% CI, 0.95-2.16), indicating systematic underestimation of risk in these high-risk strata despite acceptable performance in the aggregate cohort.

### Multivariable Regression Analysis

In multivariable logistic regression, in the entire cohort, greater STAT category was strongly associated with increased odds of 30-day mortality (OR, 2.26; 95% CI, 1.94-2.64, *P* < .001). Neonatal status was independently associated with greater baseline mortality risk (OR, 3.88; 95% CI, 1.60-9.40, *P* = .003). The interaction term between STAT and neonatal status suggested an attenuation of the effect of STAT on mortality in neonates (OR, 0.80; 95% CI, 0.63-1.01, *P* = .06).

**Figure 4:**
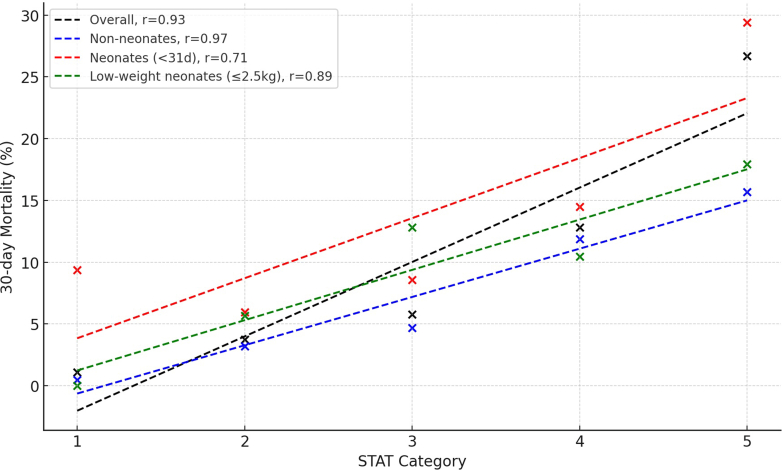
Correlation between STAT mortality category and 30-day mortality across subgroups. This figure illustrates the linear association between STAT category and observed 30-day mortality in the overall cohort and in 3 prespecified subgroups: non-neonates, neonates (<31 days), and neonates with low weight (≤2.5 kg). Across the entire population, mortality shows a strong monotonic rise with increasing STAT level (r = 0.93), driven predominantly by the high correlation observed in non-neonates (r = 0.97). In contrast, neonates exhibit a weaker correlation (r = 0.71), reflecting greater baseline physiological vulnerability that diminishes the explanatory contribution of procedural complexity alone. Neonates with low weight display an intermediate pattern (r = 0.89), with consistently elevated mortality at all STAT categories. Together, these correlations highlight the attenuation of STAT-based risk discrimination in physiology-dominated neonatal strata. *STAT*, Society of Thoracic Surgeons–European Association for Cardio-Thoracic Surgery Mortality Categories.

Low weight (<2.5 kg) was not an independent predictor of mortality after adjustment (OR, 1.54; 95% CI, 0.30-7.85, *P* = .60), and the STAT × low weight interaction was likewise not significant (OR, 0.82; 95% CI, 0.54-1.25, *P* = .35). Furthermore, on Pearson correlation analysis, STAT category was positively and strongly correlated with 30-day mortality across the entire cohort (r = 0.93). When analyzed by age group, this association appeared weaker in neonates (r = 0.71) and in neonates of low weight (r = 0.89). The strongest positive correlation between STAT category and mortality at 30 days was in the non-neonate group (r = 0.97), as presented in Figure 4.

In non-neonates, STAT showed good discrimination for 30-day mortality (AUC, 0.74; 95% CI, 0.71-0.77; Brier score 0.05), with near-ideal calibration (intercept 0.55, slope 1.05). In neonates, discrimination was attenuated (AUC, 0.66; 95% CI, 0.62-0.70), with greater overall prediction error (Brier score 0.12) and evidence of calibration drift, characterized by a positive intercept (0.16) and a slope <1 (0.72), consistent with underestimation of risk at greater predicted probabilities and overconfident predictions at the extremes. In patients with low weight, model performance was modest (AUC, 0.65; 95% CI, 0.54-0.75; Brier score 0.08), with wide CIs and an attenuated calibration slope (0.61), reflecting imprecision and limited reliability of STAT-based risk estimation in this small, high-risk subgroup.

### Analysis by Era

**Figure 5:**
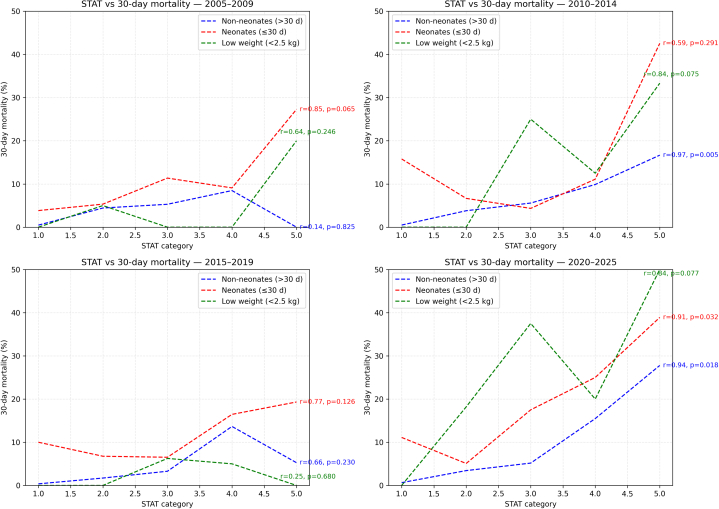
Era-stratified correlation between STAT mortality category and 30-day mortality across clinical subgroups. The figure presents 4 era-specific panels (2005-2009, 2010-2014, 2015-2019, 2020-2025), each depicting the relationship between STAT category and 30-day mortality in non-neonates, neonates (<31 days), and infants with low weight (<2.5 kg). Across all eras, non-neonates demonstrate consistently strong correlations between STAT category and mortality (r ≈ 0.90-0.97), reflecting stable predictive performance of the STAT system in older children. In contrast, neonates show weaker and more variable correlations (r ≈ 0.59-0.91), with notable attenuation during earlier eras, indicating that baseline physiologic vulnerability diminishes discrimination based solely on procedural complexity. Infants with low weight display the most inconsistent behavior (r ranging from 0.14 to 0.84), with wide variability across eras and poor correlation in several periods, underscoring the instability of STAT-based risk estimates in this high-risk subgroup. Together, these era-stratified patterns highlight that while STAT retains strong predictive value in non-neonates across time, its performance is attenuated in neonates and unreliable in infants with very low weight. These findings emphasize the influence of patient-level factors and era-related system changes on the transportability of procedure-only risk models. *STAT*, Society of Thoracic Surgeons–European Association for Cardio-Thoracic Surgery Mortality Categories.

**Figure 6:**
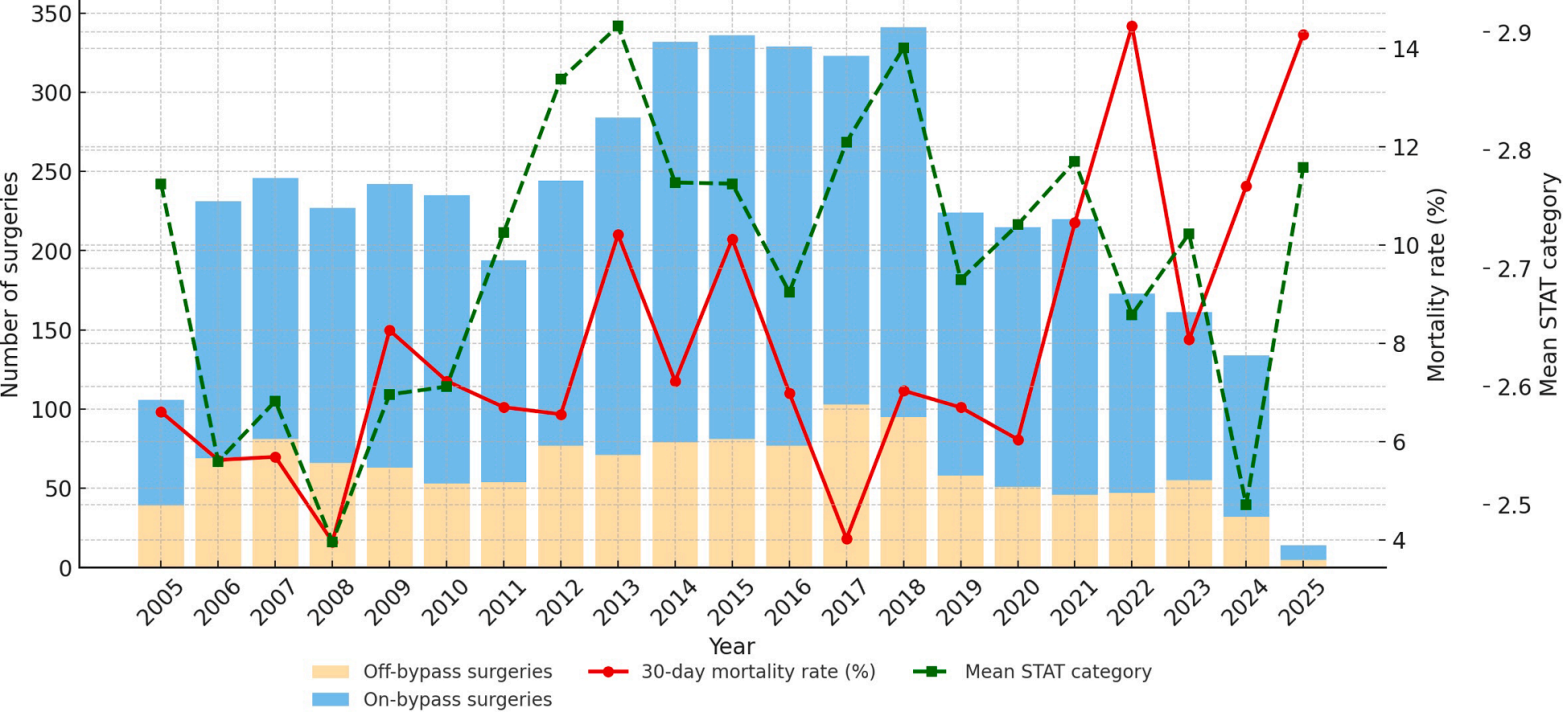
Annual surgical volume, 30-day mortality, and mean STAT category from 2005 to 2025. This figure summarizes yearly program activity over 2 decades, displaying the total number of congenital cardiac operations stratified by on-bypass and off-bypass procedures (*stacked bars*), together with annual 30-day mortality rates (*red line*) and mean STAT category (*green dashed line*). Surgical volume increased steadily after 2009, reaching a peak during 2014-2018, coinciding with expansion of surgical capacity and stabilization of referral pathways. Mean procedural complexity (*STAT*) rose gradually over time, reflecting a growing proportion of greater-risk operations. Mortality demonstrated year-to-year variability and appeared more closely aligned with changes in case mix severity—particularly increasing procedural complexity and neonatal case burden—than with absolute annual surgical volume. Transient increases in mortality coincided with periods characterized by greater proportions of complex or neonatal referrals. In the most recent years, a relative increase in mortality is observed; however, interpretation should be cautious, as outcomes in a developing program are influenced by multiple concurrent factors, including evolving case mix, changes in surgical, anesthetic, and intensive care teams, and external disruptions. The decline in surgical volume from 2023 onward corresponds to the abrupt loss of Gaza referrals after regional conflict, while overall case complexity remained relatively high. Taken together, these trends illustrate the evolving interplay between operative volume, procedural complexity, and early postoperative outcomes at a medium-volume referral center, without implying a direct causal relationship between specific programmatic events and mortality. *STAT*, Society of Thoracic Surgeons–European Association for Cardio-Thoracic Surgery Mortality Categories.

**Figure E1:**
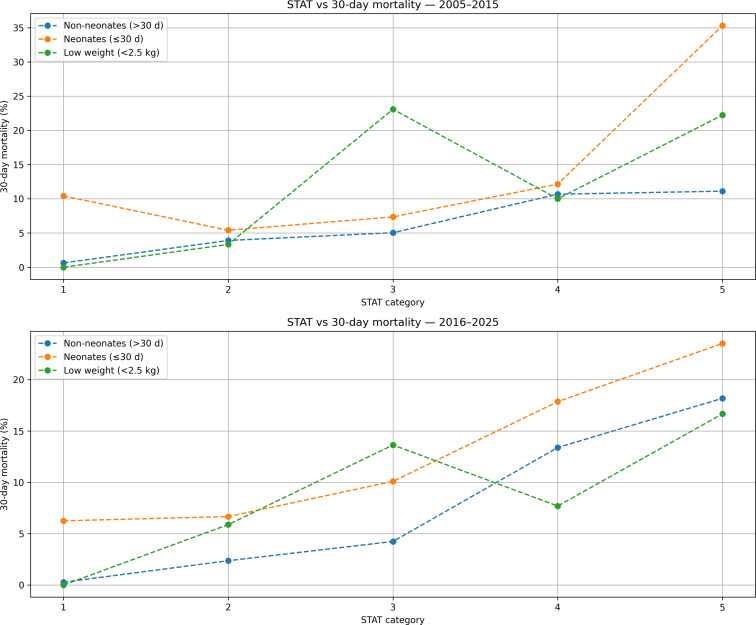
Binary era analysis of STAT categories and 30-day mortality. Relationship between STAT mortality category and observed 30-day mortality in 2 temporal eras (2005-2015 and 2016-2025). *Lines* represent mortality across STAT categories for non-neonates (>30 days), neonates (≤30 days), and infants with very low weight (<2.5 kg). The figure demonstrates that the overall pattern observed in the primary analysis remains consistent when eras are combined. *STAT*, Society of Thoracic Surgeons–European Association for Cardio-Thoracic Surgery Mortality Categories.

When stratified by era, STAT categories remained associated with 30-day mortality among non-neonates, with Pearson coefficients consistently high and logistic regression showing significant ORs per STAT increment (OR, ∼2.0-2.7, *P* < .001). Among neonates, correlation coefficients were lower and less stable (r = 0.85 in 2005-2009, r = 0.77 in 2015-2019), although logistic regression still demonstrated a significant association (OR, ∼1.5-1.7, *P* < .01). In the subgroup with very low weight (<2.5 kg), correlations were inconsistent and often not statistically significant (r = 0.84 in 2010-2014, r = 0.25 in 2015-2019), with logistic regression likewise yielding nonsignificant ORs. These findings indicate that although STAT categories maintain predictive strength across eras in older children, their discriminative ability is attenuated in neonates and particularly weak in infants with very low weight. These findings are depicted in Figure 5, whereas the temporal relation between STAT, mortality, time and procedure volume is presented in Figure 6 (see also Figure E1 and Table E1).

## Discussion

This study provides a descriptive single-center analysis of 4768 congenital cardiac operations performed over a 20-year period. Spanning more than 2 decades, mortality increased monotonically across STAT categories, confirming the broad validity of procedure-based risk stratification.^5^ However, discrimination and calibration were attenuated among neonates and infants with low weight, indicating that baseline physiologic vulnerability reduces the explanatory power of procedural complexity alone. In practical terms, who the patient is can rival what the operation is in determining early mortality risk.^14^^,^^15^ These findings suggest that although STAT performs well overall, its discriminatory performance may be attenuated in subgroups dominated by neonatal physiology and small body size.^16^ Importantly, our findings should not be interpreted as evidence of inadequacy of the STAT framework, which remains a robust and widely validated procedure-based stratification tool across large multicenter datasets, but rather as a descriptive observation highlighting how procedure-only stratification may exhibit attenuated discrimination within physiology-dominated subgroups such as neonates and infants with very low weight.

Attenuation in neonatal and low-weight strata likely reflects pathophysiologic factors not captured by procedure category: limited circulatory reserve, vessel-caliber and cannulation constraints, hemodilution during CPB, immature myocardial and renal function, and heightened susceptibility to low cardiac output, infection, and fluid shifts.^17^, ^18^, ^19^, ^20^ Neonatal mortality in “low-risk” categories—9% in STAT-1 and 6% in STAT-2—underscores this effect, highlighting how physiology can override procedural expectations even within nominally low-complexity bands. Similarly, patients with low weight exhibited particularly disproportionate outcomes: mortality in STAT-3 reached 17.1%, compared with 8.6% among neonates and 4.7% among non-neonates in the same category, a striking example of vulnerability outweighing operative complexity.^16^^,^^18^ When baseline risk is already high, the relative contribution of procedural complexity to mortality may diminish, which can lead to reduced discrimination and calibration drift when using a procedure-only stratification approach. Empirically, neonatal and low-weight groups typically demonstrate underprediction (positive calibration-in-the-large) and calibration slopes less than 1, reflecting overconfidence at both extremes of predicted risk. Nonetheless, correlation-based analyses in neonatal and low-weight subgroups should be interpreted with caution, as correlation coefficients are sensitive to smaller sample sizes, fewer observations per STAT category, and outlier events.

Despite the relatively small size of the low-weight subgroup, we elected to analyze this population separately because infants weighing <2.5 kg represent a clinically distinct and particularly vulnerable cohort in congenital cardiac surgery. Moreover, the low-weight cohort represents a physiologically extreme subset of the neonatal population, comprising approximately 15% of neonatal operations in this series. Although this group was analyzed separately to explore the potential influence of extreme body weight on procedural risk stratification, it remains a subgroup within the broader neonatal cohort. In such patients, intrinsic physiologic vulnerability related to extreme body weight may play a dominant role in determining early postoperative outcomes and may partially attenuate the relationship between procedural complexity and mortality. The analysis of this subgroup should therefore be interpreted primarily as descriptive, aimed at illustrating how procedure-based risk stratification behaves in this extreme physiologic context rather than providing definitive statistical inference. In addition, selection effects may influence the distribution of procedural complexity in infants with very low weight. In clinical practice, surgeons often defer greater-complexity repairs in infants weighing <2.5 kg and instead perform staged or palliative procedures until further physiologic maturation occurs. As a result, the observed distribution of STAT categories in this subgroup may not fully represent the natural spectrum of surgical complexity, and outcomes may therefore be driven more strongly by baseline physiologic vulnerability than by procedural risk alone.

The relationship between institutional volume and outcomes in congenital cardiac surgery has long been debated. Large-scale studies, such as those by Welke and colleagues^12^ demonstrated that outcome differences are most pronounced in high-risk procedures, and that although overall results have improved across all centers, volume-related differences persist, particularly for neonates and complex lesions.^12^ More recent work, however, highlights that volume alone is not a sufficient predictor of outcomes.^21^ Chauhan and colleagues^22^ reported the presence of “overperformers” and “underperformers” across all volume strata, whereas Anagnostopoulos and colleagues^23^ emphasized that small- and medium-volume programs can achieve outcomes close to benchmarks when supported by robust perioperative systems and coordinated care. Together, these findings suggest that outcomes are determined not only by case volume but also by infrastructure, case mix, and institutional culture.

These findings carry practical implications for benchmarking and counseling. Comparisons of O/E mortality by STAT bands demonstrate that neonatal-heavy strata frequently exceed program-level benchmarks derived from heterogeneous multicenter registries.^5^^,^^23^ This is a classic case of calibration drift: a model that performs well in aggregate can miscalibrate systematically in specific subgroups. Dashboards and intercenter comparisons that rely solely on STAT categories may therefore underestimate risk in neonatal-dense case mixes within certain clinical contexts. Presenting O/E ratios alongside calibration plots provides a clearer picture of where miscalibration occurs and how it concentrates (eg, at STAT × neonatal or weight-defined strata). Nonetheless, O/E mortality ratios should be interpreted cautiously. Greater observed mortality may reflect center-specific case mix, referral patterns, and patient-level risk factors rather than intrinsic underestimation by STAT categories. In this context, these findings are best understood as demonstrating calibration drift of procedure-only stratification in neonatal-heavy, high-risk cohorts, rather than as evidence of model deficiency. In that matter, the relatively elevated overall mortality in this cohort likely reflects an extreme case mix, including a high proportion of late-presenting and salvage patients referred after refusal by other centers. Our institutional policy of rarely declining surgical referrals, including salvage cases, likely contributes to greater observed mortality that is not captured by procedure-based risk stratification. As a result, patients undergoing procedures traditionally classified as lower STAT categories may nonetheless carry substantial physiologic risk, thereby biasing observed outcomes upward and attenuating apparent risk discrimination. In this sense, the present findings should be viewed primarily as illustrating the limits of applying procedure-only stratification within particularly vulnerable physiologic strata, rather than as evidence that the underlying STAT methodology is flawed.

Eras aligned with system changes (surgical staffing, intensive care unit organization, standardized pathways, pandemic/region-related referral shifts) help interpret performance^23^^,^^24^: era-stratified analyses and interactions (STAT × era; neonate × era) show that process improvements chiefly benefit lower-risk categories, whereas neonatal fragility remains resistant to secular gains, leaving more mortality unexplained by procedure alone (Figure 5). STAT therefore performs robustly and more stably in non-neonates across time, but less so in neonates and poorly in infants with low weight; the instability of low-weight correlations underscores the need to augment procedure-only risk with patient-level variables (eg, weight, age in days, prematurity).^14^^,^^15^^,^^18^ In our cohort, these patterns were quantitatively evident: discrimination was good in non-neonates (AUC, 0.74) with O/E ratios close to unity, whereas neonates demonstrated attenuated discrimination (AUC, 0.66) and marked underestimation of risk (O/E 1.98). Infants with low weight showed the least stable performance estimates (AUC, 0.65; O/E 1.47 with wide CIs), underscoring the limited transportability of a procedure-only metric in this high-risk subgroup. Incorporating era effects improves interpretability, mitigates temporal confounding, and enables fairer benchmarking, especially for centers with neonatal-heavy mixes in which reliance on STAT alone may misrepresent performance.^25^ Medium-volume centers with neonatal referral missions worldwide face similar calibration drift; although the transportability of our findings is likely, local calibration should be verified. Logical next steps are multicenter subgroup validation focused on neonates and infants with very low weight, and local model updating.^26^

These findings should be interpreted in the context of the intended use of STAT. STAT categories provide procedure-based stratification and facilitate transparent reporting across risk bands, but they are not intended to function as stand-alone patient-level risk adjustment. Accordingly, the STS Congenital Heart Surgery Database has established and continuously updated multivariable operative mortality risk models that incorporate procedure risk (including STAT-related information) together with patient-level factors to support fairer benchmarking and center-level comparisons. In this framework, “augmentation” is not a novel concept but rather a core principle of contemporary STS risk adjustment. These results specifically highlight that, in physiology-dominated strata such as neonates and infants with low weight, reliance on procedure-only stratification can exhibit attenuated discrimination and calibration drift. From a practical standpoint, local dashboards and counseling tools—particularly in settings where full STS model implementation is not available—may benefit from explicitly pairing STAT category with a small set of readily available patient-level variables to improve bedside interpretability. Importantly, such comprehensive STS risk adjustment models already exist, are methodologically robust, and represent the appropriate standard for risk-adjusted assessment of congenital heart surgery outcomes; broader and more consistent use of these models is essential, particularly when evaluating neonatal and other high-risk populations. This should be embedded in quality dashboards and used prospectively to refine family counseling, program benchmarking, and perioperative strategy selection.

From the perspective of a medium-volume referral center, our results also underscore the importance of case mix complexity.^12^ Over 2 decades, our program has treated a disproportionate number of neonates and international referrals, including salvage and complex redo cases, often after suboptimal previous interventions. Such populations are not fully represented in multicenter registries from which STAT was derived, and their inclusion likely explains why our observed mortality exceeds published benchmarks at the lower STAT categories. These contextual factors must be acknowledged in fair performance assessment across centers.

Toward improving risk assessment, from a clinical perspective, STAT-only risk is insufficient for family counseling in neonatal cases^14^; risk estimates should condition on patient-specific characteristics (eg, STAT × neonatal status, weight quantiles, and anticipated CPB exposure).^15^ For quality improvement, the signal points toward neonatal-focused strategies, including perfusion techniques that minimize hemodilution in infants who are very small, standardized early-extubation bundles, renal-protection measures, strict temperature and glucose control, and proactive extracorporeal membrane oxygenation readiness for high-risk strata.^26^ Finally, program dashboards should routinely stratify outcomes by both STAT and patient-level factors rather than by STAT alone, ensuring fairer benchmarking and more actionable quality metrics.^1^^,^^10^

This study has some important limitations. As a single-center analysis, generalizability is inherently constrained, and our referral pattern—heavy with international and salvage cases—may bias outcomes. Although the dataset spans nearly 20 years and includes almost 4800 operations, the sample size of infants of very low weight remains modest, widening CIs and limiting subgroup inference. Subgroup analyses in neonates and infants of very low weight are limited by smaller sample sizes and fewer events per STAT category, leading to wider CIs and reduced stability of correlation estimates. These findings should therefore be interpreted in conjunction with discrimination and calibration metrics rather than correlation alone. Analyses involving this subgroup are therefore best considered descriptive and hypothesis-generating. In addition, the low-weight cohort represents a subset of the neonatal population. Consequently, estimates within this subgroup may be sensitive to small changes in event counts. With larger sample sizes, a clearer relationship between procedural complexity and outcomes might emerge, and the findings related to this subgroup should therefore be interpreted as descriptive and hypothesis. Practice patterns evolved substantially over the study period, including changes in surgical staffing, intensive care unit organization, and referral sources, which may confound time-trend analyses.

Several methodologic considerations should be acknowledged. First, mortality definitions vary across the congenital heart surgery literature and registries. We elected to use a 30-day all-cause mortality end point inclusive of in-hospital deaths beyond 30 days, which we believe provides a comprehensive and clinically meaningful measure of early postoperative risk but which differs from the composite operative mortality definition used by the STS Congenital Heart Surgery Database. Second, because of the retrospective nature of our dataset, we were unable to fully reconstruct the detailed STS primary-procedure adjudication rules for all combined operations. Our assignment of the greatest STAT category in multiprocedure operations may therefore differ from STS classification and should be interpreted accordingly. In addition, another methodologic consideration relates to the retrospective assignment of STAT categories: all procedures were mapped to the most recent published STAT classification; however, because the study spans 2 decades, some operations performed earlier in the study period may have been categorized differently under earlier versions of the STAT system. This retrospective harmonization may introduce a degree of misclassification and should therefore be considered when interpreting the results. Related to that, Because the analysis was performed at the level of surgical procedures rather than individual patients, repeated staged operations within the same patient were treated as independent observations.

In regard to O/E analyses, these relied on STS benchmark mortality rates derived from specific reporting periods^5^ and were applied uniformly across our 20-year cohort; temporal differences between benchmark derivation and local case eras may therefore influence O/E estimates. Finally, our analysis was limited to in-hospital and 30-day mortality, without incorporating longer-term survival or morbidity endpoints such as neurodevelopmental outcomes.

## Conclusions

STAT categories remain useful for procedure-based risk stratification in congenital cardiac surgery. However, in physiology-dominated subgroups such as neonates and infants with very low weight, their discriminatory performance may be attenuated and therefore should not be interpreted as a stand-alone patient-level risk adjustment tool for center comparisons. For benchmarking, counseling, and quality improvement in these populations, reliance on procedure-only stratification may be insufficient.

Contemporary STS risk-adjustment approaches, which integrate procedure risk with patient-level factors, offer a more comprehensive framework for evaluating outcomes and communicating risk. The present findings should therefore be interpreted as descriptive and hypothesis-generating observations that highlight opportunities for complementary patient-level risk refinement rather than as a challenge to the validity of the STAT categorization itself.

### Declaration of Generative AI and AI-Assisted Technologies in the Writing Process

During the preparation of this work, the authors used ChatGPT (OpenAI) for English-language proofreading and minor grammatical and stylistic corrections. After using this tool, the authors reviewed and edited the content as needed and take full responsibility for the final version of the manuscript.

## Conflict of Interest Statement

The authors reported no conflicts of interest.

The *Journal* policy requires editors and reviewers to disclose conflicts of interest and to decline handling or reviewing manuscripts for which they may have a conflict of interest. The editors and reviewers of this article have no conflicts of interest.

## Figures and Tables

**Table E1:** Correlation analysis across binary eras

**Era**	Non-neonates (>30 d)	Neonates (≤30 d)	Low weight (<2.5 kg)
2005-2015	r = 0.97, *P* = .006	r = 0.74, *P* = .155	r = 0.76, *P* = .134
2016-2025	r = 0.96, *P* = .009	r = 0.96, *P* = .011	r = 0.85, *P* = .071

Pearson correlation coefficients (r) and corresponding *P* values describing the association between increasing STAT category and observed 30-day mortality across the 2 eras. The results demonstrate persistent discrimination of STAT categories in the non‑neonatal population and attenuated discrimination in neonates and infants with very low weight. *STAT*, Society of Thoracic Surgeons–European Association for Cardio-Thoracic Surgery Mortality Categories.
